# Gene Duplicability-Connectivity-Complexity across Organisms and a Neutral Evolutionary Explanation

**DOI:** 10.1371/journal.pone.0044491

**Published:** 2012-09-11

**Authors:** Yun Zhu, Peng Du, Luay Nakhleh

**Affiliations:** 1 Department of Computer Science, Rice University, Houston, Texas, United States of America; 2 Graduate School of Information Science and Technology, Hokkaido University, Sapporo, Japan; Washington State University, United States of America

## Abstract

Gene duplication has long been acknowledged by biologists as a major evolutionary force shaping genomic architectures and characteristics across the Tree of Life. Major research has been conducting on elucidating the fate of duplicated genes in a variety of organisms, as well as factors that affect a gene’s duplicability–that is, the tendency of certain genes to retain more duplicates than others. In particular, two studies have looked at the correlation between gene duplicability and its degree in a protein-protein interaction network in yeast, mouse, and human, and another has looked at the correlation between gene duplicability and its complexity (length, number of domains, etc.) in yeast. In this paper, we extend these studies to six species, and two trends emerge. There is an increase in the duplicability-connectivity correlation that agrees with the increase in the genome size as well as the phylogenetic relationship of the species. Further, the duplicability-complexity correlation seems to be constant across the species. We argue that the observed correlations can be explained by neutral evolutionary forces acting on the genomic regions containing the genes. For the duplicability-connectivity correlation, we show through simulations that an increasing trend can be obtained by adjusting parameters to approximate genomic characteristics of the respective species. Our results call for more research into factors, adaptive and non-adaptive alike, that determine a gene’s duplicability.

## Introduction

Gene duplication is a major evolutionary event that shapes genomic diversification across all forms of life. Consequently, it has been widely studied and its role in evolution has been investigated for a long time, particularly since Ohno’s seminal work [1]. Fueled by the large amounts of genomic and interactomic data, more analyses have been conducted and more models have been developed for gene duplication; see [2] for an excellent collection of articles in this area.

Analyses of molecular interaction data (networks) from several organisms has established a central role for gene duplication and loss in network evolution, and showed that the core group of unchanged nodes is very small [3]. However, the mechanisms and processes by which genetic networks are established are far from clear. Two areas of investigation into gene duplication can be identified, and we believe both are central to understanding network evolution and the role of gene duplication in it. The first area concerns the fate of duplicated genes [4]–[6]. Issues explored in this area include, for example, whether duplicated genes are maintained as unchanged, lose their function, undergo subfunctionalized [7], or develop new functions [8]. Other issues relate to probabilities, timings, and rates of duplication events [9]–[11], and how a new function arises in the first place [12]–[14]. A special case in this area is the fate of whole genome duplication (WGD) or segmental duplications [14], [15]. The second area concerns the preservation of duplicated genes and the role [15]–[19] as well as the role of gene duplication in adaptation [20]–[26].

Computational investigations into the evolution of molecular interaction networks have focused on graph transformation techniques for simulating how networks evolve and diversify. Using these techniques, for example, it has been shown that many biological networks exhibit scale-free characteristics and that scale-free networks can evolve through preferential attachment [27]. Additionally, it is widely accepted in this community that the most frequent genetic event resulting in node addition is gene duplication (for prokaryotic organisms, horizontal gene transfer plays a similar role to gene duplication in terms of adding genes to the genome or interactome of the host organism [28]). Consequetly, graph-theoretic models of network growth have been proposed based on gene duplication, such as the duplication-attachment (DA) models [29] and duplication-divergence (DD) models [30]–[32]. Further, others hypothesize that *link dynamics* is the dominant evolutionary force shaping the structural properties of networks, while the slower gene duplication dynamics mainly affects its size [33]. While devising models of molecular interaction networks has significant implications, e.g., ancestral network reconstruction [34]–[36], a salient feature of all these existing models is that they neither take the genomic context of the network nor do they operate in a population setting. That is, these models do not reflect how evolution truly happens: changes occur in an individual in the population, and the fate of that change is determined by adaptive (selection) and non-adaptive (e.g., mutation and genetic drift) forces.

Combining protein-protein interaction network data with gene duplication data, Prachumwat and Li observed that highly connected proteins tend to have low gene duplicability (defined by the number of duplication events a gene undergoes) and that older genes tend to have higher connectivities [37]. Using data from human and mouse, Liang and Li showed that, unlike in yeast, highly connected mammalian proteins tend to have high gene duplicability [38]. These results led the authors to hypotheses about the role of gene function in its duplicability. In particular, the authors hypothesized that mammals are more robust than yeast to dosage increase caused by gene duplication and have a higher diversification in function of gene duplicates, due to their multicellularity. Further, He and Zhang studied the correlation between gene complexity (length and number of domains) and gene duplicability using yeast data [39]. They showed that, on average, duplicate genes from either whole-genomes or individual-gene duplication have longer protein sequences, more functional domains, and more cis-regulatory motifs than singleton genes. The authors hypothesized that this is a consequence of the sub-neo-functionalization process, where complex genes are more likely to be retained after duplication because they are prone to subfunctionalization and gene complexity is regained via subsequent neofunctionalization.

In this paper, we extend the analyses of [37], [38] to a group of six species from across the Tree of Life. We show an increasing in the duplicability/connectivity correlation from *E. coli* towards *H. sapiens*, which agrees with the increasing trend in genome sizes, as well as with the phylogenetic relationship. Based on these results, we hypothesize that the observed correlations can be explained using neutral evolutionary forces, without the need to invoke adaptive arguments. We confirm this hypothesis using population genetic simulations that employ a genome-interactome genotype. We further extend the analysis of [39] to the same six species, and show an almost similar correlation to that found in the original study in yeast. This result, too, calls for more investigation into whether gene function, or the fate of a gene duplication, play any role in a gene’s duplicability.

## Results and Discussion

We analyzed gene duplicability, connectivity, and complexity data obtained from six species. Here, we report on the results, our hypothesis, and the results of a population genetic simulation to test our hypothesis.

### Correlation Among Connectivity, Age, and Duplicability

To better understand the spectrum of correlations between gene duplicability and connectivity across different species, we analyzed protein-protein interaction network and gene families in six species: *H. sapien* (Hsap), *M. musculus* (Mmus), *D. melanogaster* (Dmel), *C. elegans* (Cele), *S. cerevisiae* (Scer), and *E. coli* (Ecol). The protein-protein interaction (PPI) networks of the six species were downloaded from the STRING database [40], using a confidence value greater than 400 for the interactions. Gene families were downloaded from Ensembl Genome Database [41]. The numbers of gene families and proteins in the PPI networks for the six species are shown in Table 1. We calculated the correlations between gene duplicability and connectivity of each species as follows: for each gene family, with 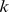 members from a species 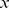, we take the average degree of the 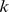 members in 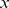
’s PPI network as the gene’s connectivity, and the size of the gene family as the gene’s duplicability. Finally, we computed Spearman’s rank correlation coefficient between duplicability and connectivity; results are shown in Table 1.

**Table 1:** Duplicability-connectivity correlations.

	Ecol	Scer	Dmel	Cele	Mmus	Hsap
Number of gene families	2906	5383	8054	10260	9247	10158
Number of genes	4258	6692	13917	20389	22791	21227
*r*	−0.138	0.081	0.172	0.221	0.224	0.290
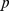 -value	10^−13^	10^−8^	10^−15^	10^−15^	10^−15^	10^−15^

Correlations between gene duplicability and connectivity in six species: *H. sapien* (Hsap), *M. musculus* (Mmus), *D. melanogaster* (Dmel), *C. elegans* (Cele), *S. cerevisiae* (Scer), and *E. coli* (Ecol). The ‘Number of gene families’ row contains, for each species, the number of gene families that had at least one member for that species. The ‘Number of genes’ row contains, for each species, the number of genes covered by the gene families. The 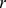 value is Spearman’s rank correlation coefficient between duplicability and connectivity, and the 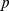-value is computed for the correlation.

We find the correlation between gene duplicability and connectivity for yeast to be positive here, though very close to 0. Prachumwat and Li reported a negative correlation between gene duplicability and connectivity. However, in their paper, they were actually looking at the relationship between gene connectivity and the proportion of unduplicated proteins. Further, the databases for gene families and PPI data have been updated since the results were obtained in [37].

Liang and Li reported a very different correlation for human and mouse from yeast in [38], and they hypothesized that the change in the correlation in different species is related to gene functions. In contrast to yeast, duplicates in mammals are more robust against a dosage increase caused by gene duplication due to the diversification in function of duplicated genes. Thus, a highly connected protein might have a better chance of survival than a duplicated non-hub protein.

**Figure 1:**
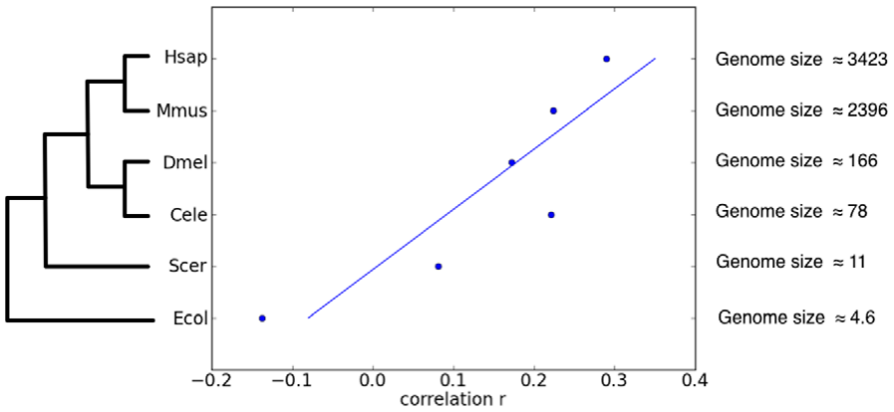
Duplicability-connectivity correlations vs. genome sizes and evolutionary relationship. Spearman’s rank correlation coefficient (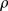) between gene duplicability and gene connectivity for six species: *H. sapien* (Hsap), *M. musculus* (Mmus), *D. melanogaster* (Dmel), *C. elegans* (Cele), *S. cerevisiae* (Scer), and *E. coli* (Ecol). The evolutionary relationship of the species is based in part on [46]. Genome size (in Mbp) information for all species, except *E. coli*, were obtained from the Animal Genome Size Database and the Fungal Genome Database.

Plotting these correlation values against genome size and evolutionary relationship information, we obtained the results in Fig. 1, which reveal an interesting trend of increasing correlation from prokaryotic organisms towards eukaryotic ones. Further, the correlation between 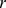 and the genome sizes of the six species is very striking (Spearman’s rank correlation coefficient of 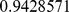 with 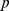-value of 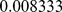).

Based on the results in Fig. 1 we hypothesize that the magnitude of neutral evolutionary forces (mutation and duplication mainly), as specific to the species or clades, might play a role in the observed correlation between duplicability and connectivity. To test this hypothesis, we conducted population genetic simulations, incorporating a genome-interactome genotype (see Methods), and inspected the correlation between duplicability and connectivity.

**Figure 2:**
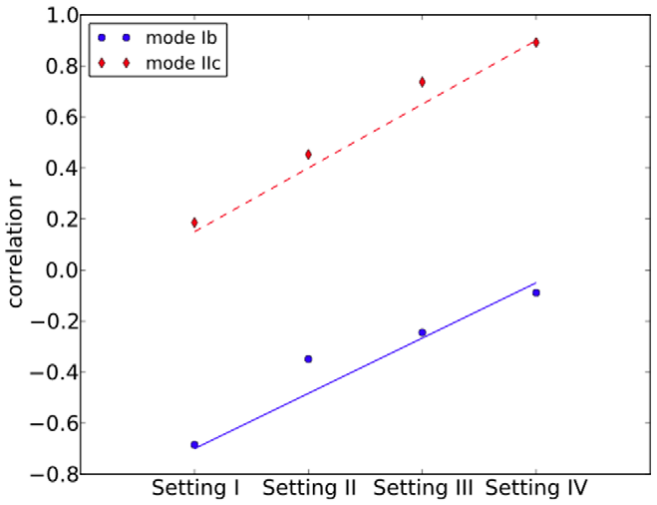
Duplicability-connectivity correlations in simulations. Spearman’s rank correlation coefficient (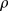) between gene duplicability and gene connectivity for different settings under the subfunctionalization model (model Ib in [5]) and the neofunctionalization model (model IIc in [5]). The parameter values in each of the four settings are given in Table 2.

In our simulations, we tested two models of gene duplication: subfunctionalization and neofunctionalization; these are models Ib and IIc, respectively, in the survey of [5] (see Methods). In the subfunctionalization model, a mutation would remove part of a gene’s function and a subset of its incident edges (interactions involving the gene). Notice that if a gene is not duplicated, then subfunctionalization will reduce the individual’s fitness and selection might consequently act to eliminate the mutant. For the neofunctionalization model, gene innovation might result in the duplicated genes gaining new edges (interactions). For each of the two models, we considered four settings, as shown in Table 2. Using other parameters (see Methods) for each setting, we ran 50 simulations, each for 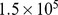 generations and calculated the average correlation between the gene duplicability and gene connectivity, as well as the average correlation between the gene age and gene connectivity of the dominant genotype (genotype whose frequency is 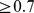). The results are shown in Table 2 and visualized in Fig. 2.

**Table 2:** Parameters and results for four simulation settings under the subfunctionalization model (model Ib in [5]) and neofunctionalization model (model IIc in [5]).

	setting I	setting II	setting III	setting IV
duplication rate	0.00001	0.000012	0.000014	0.000016
fraction of edge loss (for model Ib)	0.8	0.4	0.2	0.1
fraction of edge gain (for model IIc)	0.1	0.2	0.4	0.8
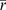 (dup vs. deg) under model Ib	−0.685	−0.349	−0.245	−0.089
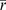 (age vs. deg) under model Ib	0.807	0.672	0.371	0.284
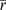 (dup vs. deg) under model IIc	0.186	0.453	0.737	0.892
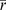 (age vs. deg) under model IIc	−0.099	−0.390	−0.613	−0.782

Fraction of edge loss indicates the number of edges that a duplicated gene loses, when it undergoes subfunctionalization, as a proportion of the number of that gene’s existing edges. Fraction of edge gain indicates the number of new edges a duplicated gene gains, when it acquires a new function, as a proportion of the number of that gene’s existing edges. The correlations are calculated by applying Spearman’s rank correlation. (p-values are less than 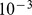.).

As the results show, we obtain an increasing trend in the correlation between duplicability and degree from Setting I to Setting IV, which are approximations of the parameter values in the different populations (Setting I is approximates a prokaryotic population, whereas Setting IV approximates a higher eukaryotic population). Notice that all correlations between gene duplicability and gene connectivity under the subfunctionalization model are negative. This is due to the fact that under this model nodes that correspond to duplicated genes tend to lose more edges than singletons (non-duplicated genes). The correlations under the noefunctionalization model, on the other hand, are much higher, which is due to the fact that gene innovation helps the duplicated genes to gain new edges.

In our simulation study, we also computed the correlation between gene age and gene connectivity. In simulation studies, and since the entire evolutionary history of the population is known, it is straightforward to estimate the age of a gene, which is the number of generations elapsed since the emergence of the gene in the population. As the results in Table 2 show, when duplicated genes (newer ones) tend to lose edges (the subfunctionalization model), there is a positive correlation between gene age and gene connectivity, and the shift in the correlation value is caused by the decrease in the fraction of edge loss. When duplicated genes tend to gain edges (the neofunctionalization model), there is a negative correlation between gene age and gene connectivity, and the shift in the correlation value is caused by the increase in the fraction of edge gain.

It is important to note that, in our simulations, genes are selected at random for duplication (based on the duplication rate), and that no selection is employed directly on duplicability or connectivity. That is, the number of times a gene duplicates and its degree in the interaction network do not affect the gene’s probability of being chosen for duplication in subsequent generations. This fact, combined with the agreement between simulation results and results from data analysis of six species, indicates that protein connectivity (which has been taken as a proxy for functional importance in other studies) may play no role in gene duplicability.

### Correlation between Gene Duplicability and Complexity

As we discussed above, He and Zhang reported a positive correlation between gene duplicability and complexity (length and number of domains) in yeast [39]. The authors hypothesized that this is a consequence of the sub-neo-functionalization process, where complex genes are more likely to be retained after duplication because they are prone to subfunctionalization and gene complexity is regained via subsequent neofunctionalization. To see whether this correlation holds for other species, we conducted similar data analysis for the six species as above; the results are shown in Table 3.

**Table 3:** Duplicability-complexity correlations.

	Ecol	Scer	Dmel	Cele	Mmus	Hsap
#families	2906	5383	8054	10260	9247	10158
#genes	4258	6692	13917	20389	22791	21227
*r* (dup vs. length)	0.234	0.137	0.137	0.183	0.240	0.255
*p*-value	10^−15^	10^−15^	10^−15^	10^−15^	10^−15^	10^−15^
*r* (dup vs. #domains)	0.232	0.133	0.270	0.282	0.379	0.325
*p*-value	10^−15^	10^−15^	10^−15^	10^−15^	10^−15^	10^−15^

Correlations between gene duplicability and length and between gene duplicability and number of domains, in six species: *H. sapien* (Hsap), *M. musculus* (Mmus), *D. melanogaster* (Dmel), *C. elegans* (Cele), *S. cerevisiae* (Scer), and *E. coli* (Ecol). The numbers of gene families and genes for each of the six species are the same as in Table 1. The 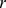 value is Spearman’s rank correlation coefficient between duplicability and connectivity, and the 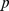-value is computed for the correlation.

Our results show that a positive correlation between gene length and gene duplicability as well as between domain numbers of a gene and gene duplicability. These results call into question the role of the sub-neo-functionalization process in explaining the emerging correlations. Given that gene duplication is a random event, and that its rate is often assumed to be constant across all genomic regions, the simplest possible explanation for a correlation between length and duplicability can be that the longer a gene (or, more generally, genomic region) is, the more duplication events would hit it. Unlike the correlation between duplicability and degree, the correlation here is fairly constant across all species, making this simple explanation the more plausible. The same trend holds for the number of domains in a gene. Our simulation framework currently does not incorporate information on gene length and numbers of domains, as the interplay between such data and other genotypic and phenotypic features is not known. Nonetheless, these analysis further underline the significance of comparative analyses to elucidate correlations, or lack thereof, among the various biological features.

## Methods

In this section, we provide details of the simulations we conducted, the various parameters that control simulations, and the parameter settings we used, based on estimates derived from the literature. We implemented nine different models of the fate and function of gene duplicates, which were surveyed in [5], and are reproduced, for ease of reference, in Table 4.

**Table 4:** Nine models of gene duplication; reproduced from [5].

Model	Description	Mutation	Fitness
Ia	Extra copies of a gene are redundant andcan be relieved from purifying selection	pseudogenization and very rare new functionalization	maintained at 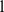 if each gene has at least one functioning copy
Ib	Each gene has subfunctions; functionally complementary copies produce one function	mutation removes a subfunction or whole function	same as Ia, with complementary copies treated as a functioning copy
Ic	functionally complementary copies canspecialize and be more advantageous	same as Ib	specialized copy has increased fitness value
IIa	Extra copies are always beneficial	same as Ia	increase in dosage results in increase in fitness
IIb	Extra copies can shield genesagainst deleterious mutations	same as Ia; simulated with a highermutation rate	same as Ia
IIc	Gene duplication develops amodified function	mutation can introduce new functionsto the extra copies	new functions increase fitness
IIIa	Original gene carries multiple subfunctions whichcan adapt to full-fledged functions in extra copies	mutation can adapt the subfunction to full function in extra copies	extra new full function increases fitness
IIIb	Different allele types pre-exist in population;duplication and recombination togethercan create advantageous heterozygote	pseudogenization	heterozygote genes have higher fitness
IIIc	Similar to IIIc, with multi-allelic diversitybeing advantageous	pseudogenization	genes that accumulate several different alleles have higher fitness

A genotype in our simulation is a coupled genome-interactome entity, where the genes on the genome component correspond to the nodes in the interactome counterpart. We consider several mutational events:

### 

#### 1. *Gene duplication*

A gene 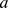 (or, set of genes) is chosen at random from the genome and is duplicated. The duplicate gene, 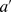, is inserted either immediately next to 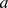 or at a random place in the genome. At the interactomic level, a new node that corresponds to gene 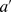 is added to the network, and is connected to all other nodes to which the node corresponding to gene 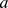 is connected.

#### 2. *Gene deletion*

A gene 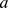 (or, set of genes) is chosen at random from the genome and is physically removed from the genome. The corresponding node for gene 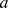, along with all edges incident with it, are removed from the network.

#### 3. *Gene mutation*

A gene may mutate and lose or partially lose its function. In this case, the function assignment to the gene is updated, and a subset of the edges (may include *all* edges) connected to the corresponding node are deleted from the network. This mode differs from gene deletion in that neither the gene in the genome nor its corresponding node in the network are removed; only their status is changed.

#### 4. *Gene innovation*

A gene may mutates to gain a new function (with or without the loss of its original function). In this case, the function assignment to the gene is updated. If the node corresponding to this gene has 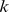 neighbors in the network, an assignment of 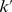 new neighbors to replace the original ones is made.

#### 5. *Gene conversion*

Given two alleles (haploid genomes) in the population, a gene 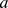 in the genome of individual 1 acquires the “status” of a corresponding gene 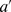 in the genome of individual 2. This event is reflected the network of individual 1 by removing all connections of gene 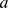 in the network, and adding connections to genes whose homologs are connected to 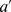 in the network of individual 2. It is important to note that while gene conversion is a special type of homologous recombination, it is simulated here in terms of two individuals in the population since our populations are haploid and random mating is assumed.

#### 6. *Recombination (cross-over)*

This event is simulated by exchanging segments of the genomes of two individuals. This exchange is reflected at the network level as follows. Interactions that involve pairs of genes on either side of a recombination breakpoint are preserved only if the interacting pair has homologs on both genomes; otherwise, such interactions are eliminated as a result of recombination.

#### 7. *Edge Addition/Deletion*

A random edge is added or removed from the network. While no changes are performed at the genome level, this operation amounts to mutations in the genes and/or regulatory regions, which, for example, affect binding sites and binding affinities, thus modifying the interactions in the network.

In this framework, pseudogenization and subfunctionalization of a duplicated gene are modeled by gene mutation. A new function may be acquired by a gene through either innovation or gene conversion.

The frequencies of genotypes in a population are governed by genetic drift (simulated by sampling individuals from the population based on the binomial distribution) and selection. In determining the fitness of an individual following a gene duplication event, we use the following principles.

1. If the duplicated gene does not acquire new function, then the individual’s fitness does not increase unless it is assumed that increase in dosage is beneficial or that the mutation rate is too high that duplicates can help shield against deleterious mutations. Likewise, the loss of a duplicate’s function does not affect fitness if another copy of the gene exists in the genome with exactly the same function as the one being lost.
2. If a gene has multiple functions and undergoes duplication, then the different copies of the gene may retain different subsets of the original set of functions. As long as the set of functions of the different copies equals the original set, no change in fitness occurs as a result of duplication. However, if maintaing a smaller subset of functions per copy improves the gene’s functioning, then duplication results in increased fitness.
3. If a duplication event results in acquiring new, additional functions, then the duplication event results in increased fitness.
4. Duplication coupled with gene conversion or recombination may result in heterozygote advantage (or diversifying advantage).

Assuming a homogeneous population at the initial generation, where a genome has 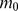 genes, each of which has a unique function, then the fitness 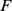 of an individual is calculated, in any generation, as
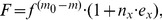
(1)where 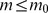 is the number of original functions (out of 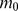) maintained in the individual, 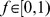 is the fitness coefficient contributed by each of the original functions. An individual may also gain new genes/functions, and the contribution of these to the individual’s fitness is given by the right term in Eq. (1). In this case, each of the 9 duplication models (Table 4) may contribute differently, and is denoted in the formula by 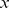, where 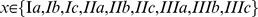 and 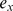 is the fitness coefficient for each of the models. For models Ia, Ib, and IIb, there is no advantage to new copies or functions; therefore, 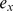 for these three models is set to 0. For model Ic, 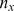 is the number of gene copies with specialized subfunctions. For model IIa, 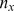 is the number of new functioning gene copies. For model IIc, 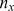 is the number of new gene copies with new functions. For model IIIa, 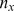 is the number of original subfunctions that are now full-fledged functions in new copies. For models IIIb and IIIc, 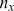 is the number of different alleles.

**Table 5:** Parameter settings used in the simulations (units for all rates are “per gene per generation”).

population size	*N* = 10^2^∼10^3^
num of generation	*n* = 10^5^∼10^6^
fitness coefficient ( *f* in Eq. (1))	*f* = 0.8
duplication fitness coefficients (*e_x_* in Eq. (1))	*e* = 0.01 (*e* = 0.001 for model IIa)
duplication rate	*d* = 10^−5^
null function mutation rate	10^−5^
edge mutation rate	10^−5^
functional innovation rate	10^−7^
gene conversion rate	*c* = 10^−5^
recombination rate	*r* = 10^−5^

Notice that if 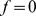, then 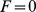 if and only if 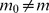. This amounts to a hard selection mechanism, where the individual is not viable whenever any of the original gene functions is lost. When 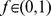, loss of original function(s) (i.e., 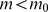) can cause fitness reduction, and the smaller the value of 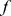 the larger the fitness reduction is. If needed, a penalty term can also be added to the fitness function 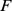 to compensate for the genome growth cost [42].

### Simulation Parameters

For our simulations, we used parameter values derived from an extensive literature survey. Forbidden (deleterious) mutations occur at an order of magnitude of 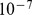 per site per generation, and the number of sites in a gene can range from 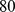 (rRNA genes) to 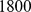 [1]. In [43], it is assumed that the duplication rate is 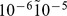 per gene per generation, that null functional mutation rate is on the order of 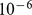 per gene per generation and that new functions arise at a rate of 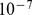 per gene per generation. In [12], the duplication rate is assumed to be 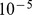 per gene per generation, null functional mutation rate is on the order of 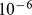 per gene per generation, and new functions arise at a rate of 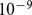 per gene per generation. In [9], the duplication rate is assumed to be 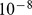 per gene per generation. These different studies also use different population sizes so that the population duplication rate (that is, the population size times the duplication rate) is much smaller than 1.

Mutation rates are often assumed to be 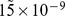 per site per generation [44]. The number of genes in a genome is on the order of 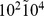. Gene conversion rate is on the order of 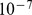 per gene per generation. The fitness coefficient (values of 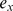 coefficients above) is widely accepted to be 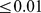. In [45], the recombination rate is estimated to be 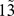 times the mutation rate. Based on these references, we used the parameter values shown in Table 5.
